# Prevention of air embolism related to central venous catheter removal: an integrative review

**DOI:** 10.1590/1677-5449.202500192

**Published:** 2025-11-07

**Authors:** Enrico Baldini Benetti, Gabriel Maraia Ciolfi, Jonatan Egian Ramos, Breno Casellatto Rodrigues Almeida, Roberta Garcia Gomes, Patrícia Scotini Freitas, Rogério Silva Lima

**Affiliations:** 1 Universidade Federal de Alfenas – UNIFAL, Alfenas, MG, Brasil.

**Keywords:** embolism, air, central venous catheter, review

## Abstract

The removal of a central venous catheter can cause serious complications, such as air embolism, but there is limited evidence on effective prevention methods. This integrative review followed PRISMA guidelines and analyzed studies in English, Portuguese, and Spanish, without time restrictions, in the LILACS, Web of Science, PubMed, CINAHL, and EMBASE databases. Two studies were selected. One compared two removal methods and found that the modified technique reduced the occurrence of air embolism compared to the traditional method. The other study indicated that removal with suture over the catheter was also effective in prevention. Despite these promising findings, experimental research is needed to determine best practices. Additionally, factors such as patient positioning and maneuvers like the Valsalva maneuver should be further studied to optimize air embolism prevention.

## INTRODUCTION

The multidisciplinary care team responsible for critical patients with instability of vital systems or risk of failure of one or more organ functions must employ specific technologies for their clinical management,^1^ including use of a venous catheter (CVC), which provide access to the patient’s systemic circulation. The jugular internal, subclavian, or femoral vein is punctured, enabling the catheter to advance as far as the proximal third of the superior vena cava, the right atrium, or the inferior vena cava, at which sites it must be correctly positioned.^2^

Central venous accesses are widely used in treatment of a huge range of patients with different clinical conditions^3^ to achieve multiple objectives, such as accessing extracorporeal blood circuits – for renal replacement therapy –, for monitoring hemodynamic variables –central venous oxygen saturation and central venous pressure–,^2^ and for administration of medications that could injure tissues if administered peripherally, such as vasopressor drugs and hypertonic solutions of sodium bicarbonate and calcium.^4^

Health professionals must receive technical training to learn how to insert and remove these types of access, considering how important they are for maintenance of patients’ treatment and the many different complications associated with insertion, dwelling, and removal. These include: bleeding, hematoma, hemothorax, hemomediastinum, pneumothorax, atrial perforation, arrhythmia, cardiac arrest, tracheal laceration, recurrent laryngeal nerve injury, incorrect placement of the catheter tip, mechanical dysfunction, thrombosis, mural thrombus, atrial thrombus, formation of a fibrin sheath, infection, central vein stenosis, and ineffective dialysis. While air embolism can occur during catheter insertion, the incidence associated with removal is greater. It is not, however, possible to determine the exact incidence or prevalence, since the majority of venous air emboli are subclinical and do not provoke observable symptoms.^5^ This complication is characterized by build up of air in the vein, causing formation of bubbles, which may or may not cause clinical manifestations, depending on the extent to which the bubbles act as a vascular embolus.^5^ Large volumes of air entering the central circulation can result in pulmonary thromboembolism, with tachypnea and dyspnea, tachycardia, and hypoxia. Furthermore, in the presence of pulmonary shunting or a patent foramen ovale, air embolism can also cause neurological injury.^6^

Pathophysiologically, the air embolus formed in the venous circulation is transported to the right side of the heart and from there to the lungs, where it can provoke obstructions causing pulmonary artery hypertension, not merely compromising gaseous exchange, but also impairing right chamber function.^7^ Moreover, an embolus may reach the arterial circulation, a condition known as paradoxical embolism, whether via the pulmonary vascular network itself, or via arteriovenous shunts, such as a patent foramen ovale,^8^ resulting in arterial air embolism, which is a critical condition associated with stroke and arrhythmia caused by reduced flow in the coronary arteries.

There is scant evidence available on prevention of pulmonary embolism during CVC removal, because of the limited availability of confirmatory studies. The article “Central venous catheter removal procedures and rationale”, published in the *British Journal of Nursing* in 2000, proposed a number of recommendations for health professionals that have been associated with reduced risk of emboli, such as: avoid performing the procedure in dehydrated patients;^9^ put the patient in the Trendelenburg position; remove the catheter while a Valsalva maneuver is performed;^10^ gently occlude the venous insertion point after removal;^9^ and maintain the patient supine for at least 30 minutes. More recent case reports also recommend application of a compressive dressing after withdrawal.^11^

While there are other reviews, such as one study^12^ dealing with CVC-related air embolism, there is still a lack of studies identifying the principal strategies for prevention of this complication during device removal and their respective effectiveness, which could be used to support clinical decision making. There is therefore a need for studies that synthesize the available evidence on safe CVC removal, with the objective of proposing improvements to clinical practice and training of health professionals, thus contributing to reduce the incidence of air embolism and the morbidity and mortality associated with it.

Considering the importance of the problem and the scenario outlined above, this integrative review was conducted with the objective of identifying the evidence available in the literature on the procedures adopted for prevention of air embolism during CVC removal in adult patients.

## METHOD

This study is an integrative literature review, which is a method for synthesis of the results of primary studies by means of an ordered and comprehensive review,^13^ with the objective of integrating their findings into clinical practice.

The integrative review was conducted in six sequential steps: formulation of the research question; literature search and selection of primary studies; classification of the studies; evaluation of the selected studies; interpretation of the results; and presentation of the review.^14^

The study was conducted in accordance with the recommendations set out in the Preferred Reporting Items for Systematic Reviews and Meta-Analyses (PRISMA),^15^ to ensure rigor in the reporting of the review . The review protocol was registered on the FigShare platform on March 5, 2024.^16^ The study was supported by the Institutional Scientific Initiation Scholarship Program (PIBIC - *Programa Institucional de Bolsas de Iniciação Científica*), funded by the National Council for Scientific and Technological Development (CNPq - *Conselho Nacional de Desenvolvimento Científico e Tecnológico*).

The patient, intervention, comparison and outcome (PICO) strategy was used to formulate the research question, as illustrated in Table 1. The question adopted was as follows: “What is the available evidence on procedures adopted for prevention of air embolism during CVC removal in adult and elderly patients?”.

**Table 1:** Components of the research question formulated using the PICO strategy, Alfenas, 2024.

**Description**	**Abbreviation**	**Components of the question**
Population	P	Patients over the age of 18 years (adults and seniors)
Intervention	I	Removal of central venous catheters
Comparison	C	Not applicable
Outcome expected	O	Prevention of air embolism

Source: The authors.

Searches were run for primary studies on March 26, 2024, in following sources: PubMed, LILACS, EMBASE, CINAHL, and Web of Science Controlled vocabulary keywords specific to each source were employed: from the Medical Subject Headings (MeSH) for PubMed and Web of Science; from the Health Science Descriptors (DeCS) for LILACS, in English, Portuguese, and Spanish; from the CINAHL Subject Headings for CINAHL; and from Emtree for EMBASE.

Primary studies published in English, Portuguese, or Spanish, with no date limits, that answered the research question were selected. The keywords and search strategies are shown in Tables 2 and 3, respectively. The search strategy was developed with the support of a librarian.

**Table 2:** Controlled and uncontrolled keywords, Alfenas, 2024.

**Database**	**Controlled keywords**	**Uncontrolled keywords**
PubMed (MeSH)	Central Venous Catheters	Central Venous Catheter
	Embolism, Air	Air Embolism
		Air Embolisms Gas embolism
		Gas embolism
LILACS (DeCS)	*Cateteres Venosos Centrais*	Central Venous Catheter
	Central Venous Catheters	*Embolia Gasosa*
	*Catéteres Venosos Centrales*	*Embolia de Ar*
	*Embolia Aérea* Embolism, Air	Air Embolism
	*Embolia Aérea*	Air Embolisms Gas embolism Gas embolism
		*Embolia Gaseosa*
		*Embolia de Aire*
	
EMBASE (Emtree)	central venous catheter	AXERA
	air embolism	Broviac
		central intravenous
		catheter
		central line
		central vein catheter
		central venous access catheter
		central venous access device
		central venous catheters
		central venous line
		cv cath
		CVP line
		Groshong
		Leonard
		Leonard catheter
		LOGICATH
		Orion II
		PediaSat
		Powerwand
		Pro-Line
		Secalon-T
		short-term central venous
		catheterization kit
		Vortex Port aero-embolism
		aeroembolism
		air embolus
		embolism air
CINAHL (CINAHL Subject Headings)	Embolism, Air	Air Embolism Gas embolism Broviac Catheter Broviac Catheters
	Central Venous Catheters	Catheter Central Venous Central Venous Catheter Hickman Catheter Hickman Catheters
Web of Science (MeSH)	Central Venous Catheters Embolism, Air	Central Venous Catheter
		Air Embolism
		Air Embolisms Gas embolism Gas embolism

Source: The authors.

**Table 3:** Integrative review search strategy, Alfenas, 2024.

**Database**	**Search strategy**
PubMed	((((((“Central Venous Catheters”) OR (“Central Venous Catheter”)) AND (“Embolism, Air”)) OR (“Air Embolism”)) OR (“Air Embolisms”)) OR (“Gas embolism”)) OR (“Gas embolism”)
LILACS	(“*Cateteres Venosos Centrais*”) OR (“Central Venous Catheters”) OR (“Central Venous Catheter”) OR (“*Catéteres Venosos Centrales*”) AND (“*Embolia Aérea*”) OR (“*Embolia Gasosa*”) OR (“*Embolia de Ar*”) OR (“Embolism, Air”) OR (“Air Embolism”) OR (“Air Embolisms”) OR (“Gas embolism”) OR (“Gas Embolism
	Gas Embolisms
	“) OR (“*Embolia Aérea*”) OR (“*Embolia Gaseosa*”) OR (“*Embolia de Aire*”)
EMBASE	('central venous catheter' OR 'axera' OR 'broviac' OR 'central intravenous catheter' OR 'central line' OR 'central vein catheter' OR 'central venous access catheter' OR 'central venous access device' OR 'central venous catheters' OR 'central venous line' OR 'cv cath' OR 'cvp line' OR 'groshong' OR 'leonard' OR 'leonard catheter' OR 'logicath' OR 'orion ii' OR 'pediasat' OR 'powerwand' OR 'pro-line' OR 'secalon-t' OR 'short- term central venous catheterization kit' OR 'vortex port') AND ('air embolism' OR 'aero-embolism' OR 'aeroembolism' OR 'air embolus' OR 'embolism air') AND [embase]/lim
CINAHL	(“Embolism, Air” OR “Air Embolism” OR “Gas embolism”) AND (“Central Venous Catheters” OR “Broviac Catheter” OR “Broviac Catheters” OR “Catheter Central Venous” OR “Central Venous Catheter” OR “Hickman Catheter” OR “Hickman Catheters”)
Web of Science	“central venous catheters” (Topic) OR “central venous catheter” (Topic) AND “embolism, air” (Topic) OR “air embolism” (Topic) OR “air embolisms” (Topic) OR “gas embolism” (Topic) OR “gas embolism” (Topic)

Source: The authors.

Results were organized using EndNote^17^ and Rayyan.^18^ After exclusion of duplicate search results, studies were selected by two reviewers independently, in two stages: first, titles and abstracts were read and articles answering the research question were selected; then, the full texts of articles were read. A third reviewer was consulted in cases in which there was no consensus on article eligibility. Finally, the references of the studies selected were examined to identify possible additional articles.

Data were extracted following a roadmap developed by the authors, structured in the form of a synthesis table (Table 4) for each study included in the integrative review. This table was used to record title, authors, journal, country, data, study design, evidence level, objective, method, results, and conclusions.

**Table 4:** Synthesis of included studies, Alfenas, 2024.

**Title**	**Authors, journal, country, data**	**Study design, evidence level**	**Objective**	**Method**	**Results**	**Conclusions**
Tunnelled haemodialysis catheter removal: an underappreciated problem, not always simple and safe^22^	Tomasz Porazko, Jacek Hobot, Zbigniew Ziembik, and Marian Klinger, *International Journal of Environmental Research and Public Health*, Poland, April 27, 2020.	Retrospective study, evidence level 4	To analyze use of the modified cut-down method (MCDM) for removal of central venous catheters.	Retrospective analysis of the medical records of 143 patients at the Department of Nephrology and Dialysis and the Department of General and Vascular Surgery of the Opole University Hospital, with comparison of results of patients who underwent the cut-down method (CDM) or the MCDM.	One case of air embolism was observed related to the CDM technique, which was treated successfully. There were no cases of air embolism among patients who underwent the MCDM technique.	The MCDM technique proved safer for prevention of air embolism in removal of central venous catheters.
Over-catheter tract suture to prevent bleeding and air embolism after tunnelled catheter removal^23^	Krzysztof Letachowicz, Tomasz Gołębiowski, Mariusz Kusztal, Jan Penar, Waldemar Letachowicz, Wacław Weyde, and Marian Klinger, *Journal of Vascular Access*, Poland, March 21, 2016	Retrospective study, evidence level 4	To demonstrate that the catheter removal technique with over-catheter suture offers efficacy for prevention of air embolism and bleeding during removal of central venous catheters.	Evaluation of the effectiveness of a technique for removal of central venous catheters, by analysis of the medical records of 79 patients who underwent the procedure at a hospital in Wroclaw, Poland.	The catheter removal technique with over-catheter suture demonstrated greater safety for prevention of air embolism compared to the conventional technique.	Use of the catheter removal technique with over-catheter suture reduced the risk of air embolism during removal of central venous catheters.

Source: The authors.

Data were extracted by two reviewers independently. Studies’ evidence levels were rated following Melnyk & Fineout-Overholt,^19^ and their methodological quality was rated using the McMaster University Occupational Therapy Evidence-Based Practice Research Group criteria, in accordance with the study designs of the included studies.^20^

## RESULTS

**Figure 1:**
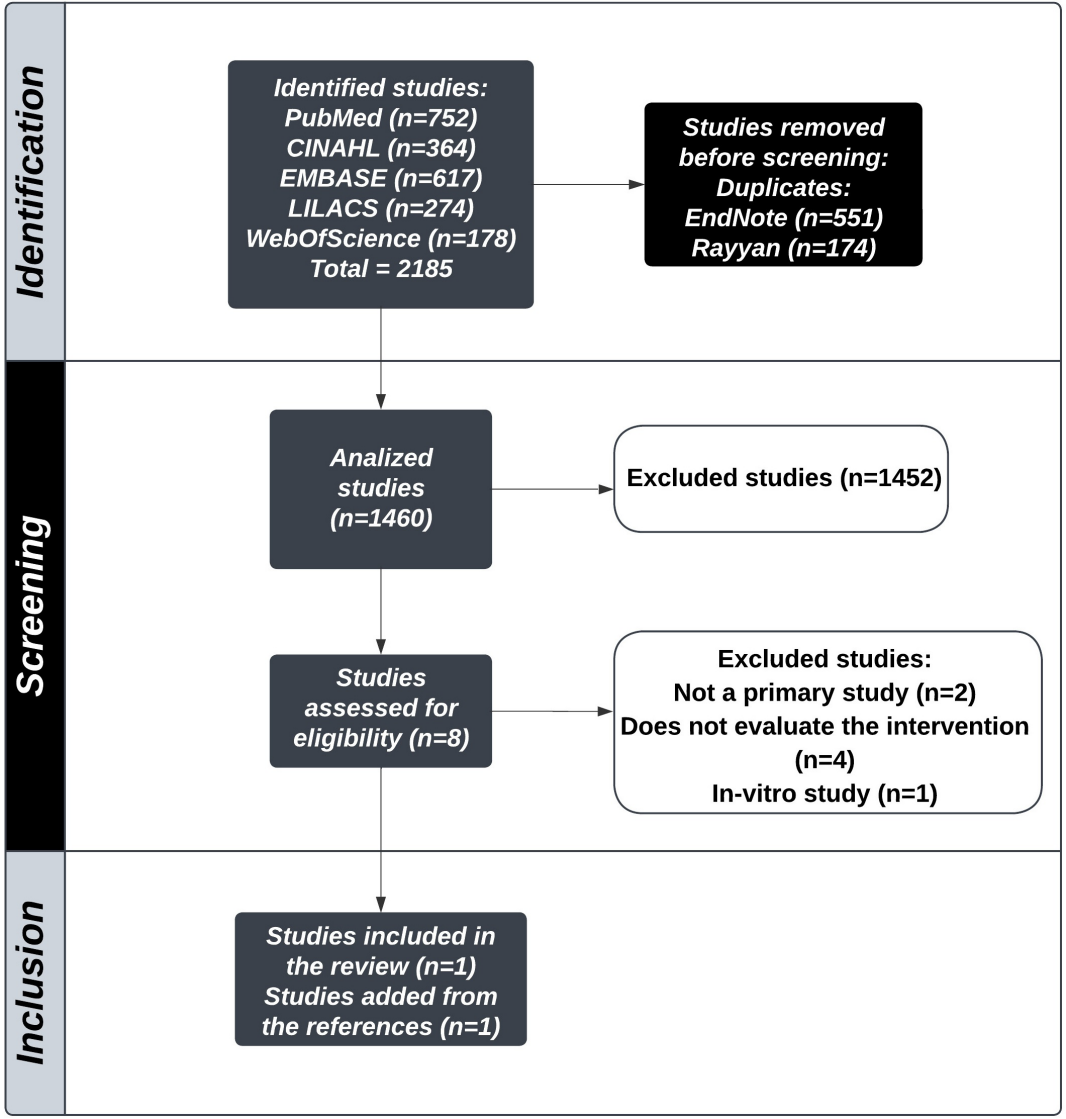
PRISMA flow diagram. Source: adapted from Page et al.^15^. PRISMA = Preferred Reporting Items for Systematic Reviews and Meta-Analyses.

The search strategy returned 2,185 studies, 752 from PubMed, 364 from CINAHL, 617 from EMBASE, 274 from LILACS, and 178 from Web of Science. After application of the inclusion criteria, two articles were selected for the final sample, as illustrated in the PRISMA flow diagram^21^ (Figure 1). The synthesis of these two studies is shown in Table 4.

In 2020, a retrospective study was conducted in Poland with the objective of evaluating a modified CVC removal method– the modified cut-down method (MCDM).^22^ The study population comprised patients from the Department of Nephrology and Dialysis and the Department of General and Vascular Surgery at the Opole University Hospital. The study method was a retrospective analysis of 76 medical records of patients who underwent the standard cut-down method (CDM) – and 67 medical records from patients who underwent the MCDM, followed by a comparison of the techniques. The results revealed one case of air embolism in the group of patients who underwent the CDM technique and zero cases of the complication in the group who underwent the MCDM.

A retrospective study conducted in Poland and published in 2016^23^ assessed a new method – the catheter removal technique with over-catheter suture. The study population comprised 79 patients from a hospital in Wroclaw, Poland. The study method was a retrospective analysis of 40 medical records from patients who underwent the conventional technique and 39 medical records from patients who underwent the new technique, followed by comparison of the two groups. The results revealed that the new technique achieved greater efficacy for prevention of air embolism after catheter removal, in addition to being simpler to perform and not involving additional costs.

Table 5 shows the comparisons between the techniques described in the two studies included in this review. Table 6 shows the results of the study quality assessments conducted using the criteria from the McMaster University Occupational Therapy Evidence-Based Practice Research Group tool.

**Table 5:** Comparison of the techniques proposed in the included studies, Alfenas, 2024.

**Steps**	***Modified cut-down method***	***Catheter removal technique with over-catheter suture***
Patient positioning	Trendelenburg	Mild Trendelenburg
Asepsis	Use of sterile technique, with no details provided	Disinfection of the area and use of sterile fields
Anesthesia	Injection of local anesthetic (lidocaine 1%) along the incision site	Injection of local anesthetic along the suture area and close to the cuff of the catheter
Suture (before catheter removal)	Not used	A suture is placed around the catheter as close as possible to the point of catheter entry into the internal jugular vein.
Incision	2 cm incision parallel to the catheter	Small incision close to the cuff
Catheter removal	Tissues are dissected and the catheter is isolated. Next, without sectioning the catheter, the intravenous part is withdrawn from the superior vena cava via the cutaneous incision. Pressure is applied over the point of vein entry for some minutes. After removal, the catheter is sectioned distal of the cuff and the distal part is removed via the exit site.	The catheter is isolated and exposed with the aid of surgical forceps. The cuff is dissected and the intravenous part is sectioned. Next, this part is removed while the patient holds his or her breath.
Hemostasis	Hemostasis is applied.	Not mentioned
Synthesis	The incision is closed with 2 to 3 non-absorbable 3.0 sutures	Non-absorbable suture along the catheter tract, while light pressure is applied to the internal jugular vein. Additional sutures are placed at the site from which the cuff was removed.
Dressing	After closure, a dressing is applied over the surgical wound. The time until removal of sutures is not specified.	After closure, the surgical site is disinfected again and a dressing is applied over the wound. The sutures are removed after 72 h.

Source: The authors.

**Table 6:** Rating the methodological quality of the quantitative studies, Alfenas, 2024.

**Critical review of the quantitative studies**		**Study 1** ^22^	**Study 2** ^23^
Study Purpose:	Does the article clearly state its purpose? (yes/no)	Yes	Yes
Literature review	Was the relevant literature on the subject reviewed?	No	Yes
Study design	Randomized/cohort/single case design/before and after/ case-control/cross-sectional/case study/longitudinal	Yes	Yes
Sample	Was the sample described in detail? (yes/no)	Yes	No
Sample	Was sample size justified? (yes/no/not applicable)	No	No
Outcomes	Were the outcome measures reliable? (yes/no/not addressed)	Yes	Not addressed
Outcomes	Were the outcome measures valid? (yes/no/not addressed)	Yes	Yes
Intervention	Intervention was described in detail? (yes/no/not addressed)	Yes	Yes
Intervention	Contamination was avoided? (yes/no/not addressed/not applicable)	Yes	Yes
Intervention	Cointervention was avoided? (yes/no/not addressed/not applicable)	Not addressed	Not addressed
Results	Results were reported in terms of statistical significance? (yes/no/not applicable/not addressed)	Yes	No
Results	Were the analysis methods adequate? (yes/no/not addressed)	Yes	Not addressed
Results	Clinical importance was reported? (yes/no/not addressed)	Yes	Yes
Results	Drop-outs were reported? (yes/no)	No	No
Conclusions	Conclusions were appropriate given study methods and results? (yes/no)	Yes	Yes

Source: The authors.

## DISCUSSION

This review found a dearth of studies documenting the effectiveness of CVC removal techniques and was able to analyze just two articles, both conducted in Poland, with retrospective designs and evidence level four, focused on removal of long-dwelling catheters.^22,23^ This scenario underscores the need for new studies with experimental designs and conducted in clinical settings.

The two studies included in this review document different techniques for CVC removal, targeting prevention of air embolism. The MCDM technique, described in 2020, was a modification of the conventional method (CDM).^19^ In the MCDM, an incision is made and tissues are dissected to locate and release the cuff of the tunneled catheter. The intravenous part of the catheter is then removed, with hemostasis by local compression, and then a cut is made above the cuff. Finally, the catheter extremity is withdrawn via the original catheter insertion orifice, followed by sutures and dressing. The difference in relation to the CDM is that in the MCDM, the incision above the cuff is made before withdrawal of the intravenous portion. According to the study, there was one case of air embolism (1.3%) in the group that underwent CDM, whereas this complication did not occur in the group that underwent MCDM, suggesting that the modified technique may be effective for prevention of air embolism related to CVC removal.^22^

The technique for catheter removal with over-catheter suture reported in 2016,^23^ comprises the following steps: the patient is placed in the mild Trendelenburg position, antisepsis of the catheter insertion area is performed and followed by isolation with sterile fields. Local anesthetic is administered and a suture is positioned around the internal jugular vein, at the point of catheter insertion, proximal to the cuff. An incision is then made in the skin close to the cuff, which is isolated and exposed with the aid of surgical forceps. The cuff is exposed and the catheter is clamped above it. The catheter is then cut and its intravenous segment is removed, while the patient performs apnea, by holding his or her breath. The suture that was placed earlier is then tied over the tract of the internal jugular vein, where the intravenous part of the catheter had been, with application of light manual compression. Additional sutures are placed at the point of incision, followed by further local antisepsis and application of a dressing. The sutures tend to be removed 72 hours after the procedure.

It should be noted that the investigative methods employed in these studies do not support conclusions as to the superiority of one technique over another for prevention of air embolism during CVC removal. However, no studies were found in the literature that compared the effectiveness of different techniques with greater methodological rigor, just descriptions in case reports.^24-26^

For example, one report describes the case of a 66-year-old male patient who underwent placement of a short-term, high-flow catheter in the right internal jugular vein for plasmapheresis because of acute renal injury.^24^ A hospital bed malfunction led to the patient having the catheter removed with his head slightly elevated, in the supine position. Soon afterwards, he developed severe dyspnea, hemiparesis, tachycardia, hypoxia, and hypotension and was diagnosed with cerebral air embolism. Treatment included 100% oxygen and Trendelenburg positioning, with subsequent improvement.^24^ These findings suggest that patient position is a relevant factor in prevention of air embolism, since air enters because of negative pressure in the catheter. Moreover, according to specialist opinion,^27^ this precaution, in conjunction with immediate occlusion of the orifice at the moment the catheter is removed, is recommended in the literature for averting air embolism. It should be emphasized, however, that although these case reports discuss such prophylactic measures, their evidence levels do not support generalization of results.

Another case report, published in 2023, also demonstrates the importance of patient position during CVC removal.^25^ In this report, a 77-year-old male patient admitted for perforated sigmoid diverticulitis had a low-flow, short-stay catheter removed from the right internal jugular vein while in a sitting position. Immediately after the procedure he developed arterial hypotension, reduced level of consciousness, local pain, and left hemiparesis. Imaging exams showed bubbles in the cavernous sinuses and basal cisterns; the patient was treated and the bubbles were reabsorbed. The diagnosis of retrograde air embolism secondary to central line removal underscores the importance of conducting this procedure with the patient horizontal on the bed.^25^ It should be noted, however, that no studies were found that compared which is the safest position for CVC removal.

In 2013, a case was described in which a fibrin tract formed around the catheter, which is more likely with long-dwelling catheters.^26^ The patient was a 75-year-old woman with hypertensive cardiomyopathy and episodes of heart failure and atrial fibrillation who had been admitted to an intensive care unit for monitoring after surgery to remove the right parotid gland. During admission, imaging exams showed an incorrectly positioned CVC and she was diagnosed with complete thrombotic occlusion of the left internal jugular vein, indicating that catheter removal was necessary. The patient was on mechanical ventilation and was placed in the Trendelenburg position. During the procedure, resistance to catheter removal was encountered. Immediately after removal local pressure was applied with gauze to control bleeding. The patient later developed hemodynamic instability. An echocardiogram showed microbubbles of air in the right cardiac chambers that were synchronous with breathing and appeared to originate in the superior vena cava. Computed tomography of the neck and chest showed total thrombotic occlusion of the left internal jugular vein, extending to the brachiocephalic trunk and left subclavian vein, with air bubbles within. A diagnosis was made of venous air embolism associated with CVC removal. A second vascular ultrasonography revealed a tract between the vein and the dermis, responsible for the air embolism and still visible more than 24 hours after placement of an air occlusive dressing.^26^

This report suggests that fibrin tracts can form around catheters, especially long-dwelling ones, increasing the risk of air embolism after CVC removal by creating a path for entry of air. As such, techniques that occlude these tracts immediately after catheter removal could reduce the incidence of air embolism associated with the procedure.

Therefore, strategies such as the over-catheter tract suture method^20^ may be useful for preventing air embolism caused by a persistent path between the dermis and the vein. It is important to consider that no studies were identified that attempt to determine best technique for dressings after catheter removal or the time required for complete occlusion of the orifice.

The article published in 2016 describes removal of a catheter with the patient holding his breath,^20^ whereas the 2020 study does not describe this intervention.^19^ While no studies were found that analyzed the effectiveness of this practice during catheter removal, a study from 2001 compared central venous pressure between different maneuvers performed during CVC insertion.^28^ Three different situations were analyzed: patient humming, holding breath, or performing a Valsalva maneuver. Central venous pressure was measured and it was observed that the Valsalva maneuver increased central venous pressure the most and resulted in the lowest percentage of negative intravascular pressure in the 40 patients assessed (2.5% = 1/40).^28^ As such, using the Valsalva maneuver during catheter removal may be an effective measure for prevention of this complication.

Given the severity of air embolism, several treatment measures have been documented in the literature,^29^ including 100% oxygenation of the patient in the Trendelenburg position, aspiration via catheter, or use of a hyperbaric chamber. Empirical antibiotic therapy with coverage for *Staphylococcus aureus* and *S. epidermidis* is also mentioned. Considering that treatment is beyond the scope of this review, it is suggested that readers consult other reviews to determine the effectiveness of these measures.

Limitations of the studies presented include the retrospective nature of the analyses and the small sample sizes. No studies were identified that analyzed removal of short-stay and low-flow catheters. Secondary studies, case reports, gray literature, letters to the editor, and editorials were excluded from the review. Despite these limitations, the studies reviewed present promising options for reduction of harm to patients, such as standardization of procedures and adoption of new techniques. Notwithstanding, the interventions proposed should consider the different sociocultural contexts and the costs incurred for provision of care during catheter removal.

## CONCLUSIONS

This review showed that there is a dearth of studies presenting the best scientific evidence on prevention of air embolism during CVC removal in adults. Two studies reported techniques that could be beneficial: the MCDM method and the catheter removal technique with over-catheter suture. However, additional experimental studies are needed to determine which technique achieves the best results for prevention of air embolism.

However, it cannot be determined which of the procedures commonly adopted in clinical practice for catheter removal constitute the most effective measures for prevention of emboli — whether placing the patient in the Trendelenburg position, performing a Valsalva maneuver, or application of compressive dressings over the removal site. Therefore, further studies are needed to assess the effectiveness of these interventions.

This review therefore demonstrates a need for greater discussion of the subject, considering that the consequences of air embolism related to CVC removal can be highly prejudicial to patients and constitute an undesired effect of health care. Moreover, the lack of standardization of catheter removal encourages use of intuitive practices, which do not ensure the safety of procedures and can lead to iatrogeny.

## Data Availability

Compartilhamento de dados não se aplica a este artigo, pois nenhum dado foi gerado ou analisado.
