# Supplementary material for: Prevention of air embolism related to central venous catheter removal: an integrative review
Source: J Vasc Bras. 2025 Nov 7;24:e20250019. doi: 10.1590/1677-5449.202500192 (PMC12642924; doi:10.1590/1677-5449.202500192)
Supplement: Translation

# Translation: Prevenção de embolia gasosa relacionada à remoção de cateter venoso central: revisão integrativa

**DOI:** 10.1590/1677-5449.202500191

**Authors:** Enrico Baldini Benetti, Gabriel Maraia Ciolfi, Jonatan Egian Ramos, Breno Casellatto Rodrigues Almeida, Roberta Garcia Gomes, Patrícia Scotini Freitas, Rogério Silva Lima

**Affiliations:** 1 Universidade Federal de Alfenas – UNIFAL, Alfenas, MG, Brasil.

**Keywords:** embolia aérea, cateteres venosos centrais, revisão

## Abstract

A remoção do cateter venoso central pode ocasionar complicações graves, como a embolia gasosa, embora existam poucas evidências sobre métodos eficazes para sua prevenção. Esta revisão integrativa seguiu as diretrizes do *Preferred Reporting Items for Systematic Reviews and Meta-Analyses*, incluiu estudos em inglês, português e espanhol, sem restrição temporal, pesquisados nas bases LILACS, Web of Science, PubMed, CINAHL e EMBASE. Foram selecionados dois estudos: um comparou dois métodos de remoção e demonstrou que a técnica modificada reduziu a ocorrência de embolia gasosa em relação ao método tradicional; o outro indicou que a remoção com sutura sobre o cateter também foi eficaz na prevenção. Apesar desses achados promissores, há necessidade de mais pesquisas experimentais para definir as melhores práticas. Além disso, fatores como o posicionamento do paciente e manobras como a de Valsalva devem ser mais investigados para otimizar a prevenção da embolia gasosa.

## INTRODUÇÃO

A assistência ao paciente grave, que apresenta instabilidade de sistemas vitais ou risco de deterioração de uma ou mais funções orgânicas, requer o emprego de tecnologias específicas para o manejo clínico pela equipe multiprofissional^1^, como a utilização do cateter venoso central (CVC), o qual representa uma via de acesso à circulação sistêmica do paciente. A punção das veias jugular interna, subclávia ou femoral permite a progressão do cateter até o terço proximal da veia cava superior, o átrio direito ou a veia cava inferior, locais onde deve permanecer adequadamente posicionado^2^.

Amplamente utilizado no tratamento de diversos pacientes com diferentes condições clínicas^3^, o acesso venoso central tem múltiplas finalidades, como possibilitar o acesso a circuitos extracorpóreos de sangue – na terapia renal substitutiva –, o monitoramento de variáveis hemodinâmicas – saturação venosa central de oxigênio e pressão venosa central^2^ – e a administração de medicamentos que podem ocasionar lesão tecidual quando administrados perifericamente, como as drogas vasopressoras e as soluções hipertônicas de bicarbonato de sódio e de cálcio^4^.

Ressalta-se a necessidade da capacitação técnica dos profissionais para a inserção e retirada desse tipo de acesso, considerando sua importância para a manutenção da terapia do paciente e as várias complicações associadas à sua inserção, permanência e retirada. Entre elas, destacam-se: sangramentos, hematomas, hemotórax, hemomediastino, pneumotórax, perfuração atrial, arritmias, parada cardíaca, laceração traqueal, lesão do nervo laríngeo recorrente, posicionamento inadequado da ponta do cateter, disfunção mecânica, trombose, trombo mural, trombo atrial, formação de bainha de fibrina, infecção, estenose de veia central e diálise inadequada. Embora a embolia gasosa possa ocorrer durante a inserção do cateter, sua incidência é maior na remoção. Não é possível, entretanto, determinar sua incidência e prevalência, uma vez que a maioria das embolias gasosas venosas são subclínicas e não produzem sintomas evidentes^5^. Essa complicação caracteriza-se pelo acúmulo de gás nos vasos venosos, levando à formação de bolhas, que podem ou não gerar manifestações clínicas, a depender do papel de êmbolo vascular que a bolha exerça^5^. A entrada de grandes volumes de ar na circulação central pode resultar em tromboembolismo pulmonar, com quadro de taquidispneia, taquicardia e hipóxia. Além disso, na presença de shunts pulmonares ou forame oval patente, os êmbolos gasosos podem ocasionar danos neurológicos^6^.

Fisiopatologicamente, o êmbolo de ar formado na rede venosa é transportado até o coração direito e, em seguida, para o pulmão, onde pode provocar obstruções que levam à hipertensão da artéria pulmonar, comprometendo não apenas a troca gasosa, mas também o funcionamento das câmaras direitas^7^. Ademais, o êmbolo pode alcançar a circulação arterial no chamado embolismo paradoxal, seja pela própria rede vascular pulmonar, seja por shunts arteriovenosos, como o forame oval patente^8^, resultando em embolia gasosa arterial, um quadro grave, associado a acidente vascular cerebral e arritmias decorrentes da redução do fluxo nas artérias coronárias.

As evidências sobre a prevenção de embolia pulmonar durante a retirada do CVC ainda são escassas, em razão da limitada disponibilidade de estudos comprobatórios. O artigo “Central venous catheter removal procedures and rationale”, publicado no *British Journal of Nursing* em 2000, apresentou algumas indicações aos profissionais, associadas a menor risco de embolia, como: evitar o procedimento em pacientes desidratados^9^; posicionar o paciente em Trendelenburg; realizar a retirada durante a manobra de Valsalva^10^; ocluir gentilmente o ponto de inserção venosa após a retirada^9^; e manter o paciente deitado por pelo menos 30 minutos. Relatos de caso mais recentes também recomendam a aplicação de curativo compressivo após a retirada^11^.

Embora existam outras revisões, a exemplo de uma pesquisa^12^ que abordou a embolia gasosa relacionada ao CVC, ainda são escassos os estudos que identifiquem as principais estratégias e sua efetividade na prevenção dessa complicação durante a retirada do dispositivo, capazes de subsidiar a tomada de decisão clínica. Assim, torna-se necessária a realização de estudos que sintetizem as evidências disponíveis sobre a remoção segura do CVC, com o objetivo de propor melhorias na prática clínica e na formação dos profissionais de saúde, contribuindo para a redução da incidência de embolia gasosa e da morbimortalidade associada.

Considerando a relevância do problema e o cenário apresentado, esta revisão integrativa tem como objetivo identificar, na literatura, as evidências disponíveis sobre os procedimentos adotados para a prevenção de embolia gasosa durante a retirada de CVC em pacientes adultos.

## MÉTODO

Trata-se de uma revisão integrativa da literatura, método voltado à síntese dos resultados de estudos primários por meio de uma pesquisa ordenada e abrangente^13^, com vistas à integração dos achados à prática clínica.

A revisão integrativa foi conduzida em seis etapas sequenciais: formulação da questão de pesquisa; busca na literatura e seleção dos estudos primários; categorização dos estudos; avaliação dos estudos incluídos; interpretação dos resultados; e apresentação da revisão^14^.

Para assegurar o rigor no relato da revisão, seguiram-se as recomendações do *Preferred Reporting Items for Systematic Reviews and Meta-Analyses* (PRISMA)^15^. O protocolo da revisão foi registrado na plataforma FigShare em 5 de março de 2024^16^. Este trabalho contou com o apoio do Programa Institucional de Bolsas de Iniciação Científica (PIBIC) do Conselho Nacional de Desenvolvimento Científico e Tecnológico (CNPq).

Para a elaboração da questão norteadora, utilizou-se a estratégia *patient, intervention, comparison and outcome* (PICO), conforme ilustrado na Tabela 1. Assim, definiu-se a seguinte pergunta norteadora: “Quais são as evidências disponíveis sobre os procedimentos adotados para a prevenção de embolia gasosa durante retirada de CVC em pacientes adultos e idosos?”.

**Tabela 1:** Componentes da pergunta de pesquisa de acordo com a estratégia PICO, Alfenas, 2024.

**Descrição**	**Abreviação**	**Componentes da pergunta**
População	P	Pacientes com idade acima de 18 anos (adultos e idosos)
Intervenção	I	Remoção de cateteres venosos centrais
Comparação	C	Não se aplica
Desfecho (*outcome*) esperado	O	Prevenção de embolia gasosa

Fonte: Elaborada pelos autores.

A busca dos estudos primários foi realizada em 26/03/2024 nas fontes de dados PubMed, LILACS, EMBASE, CINAHL e Web of Science. Foram empregados descritores controlados específicos de cada fonte: *Medical Subject Headings* (MeSH) para PubMed e Web of Science; Descritores em Ciências da Saúde (DeCS) para LILACS, nos idiomas inglês, português e espanhol; *CINAHL Subject Headings* para CINAHL; e Emtree para EMBASE.

Foram incluídos estudos primários, publicados em inglês, português ou espanhol, sem restrição temporal, que respondessem à pergunta norteadora. O detalhamento dos descritores e da estratégia de busca encontra-se nas Tabela 2 e Tabela 3, respectivamente. Ressalta-se que a estratégia de busca foi elaborada com o apoio de um bibliotecário.

**Tabela 2:** Descritores controlados e não controlados, Alfenas, 2024.

**Base de dados**	**Descritores controlados**	**Descritores não controlados**
PubMed (MeSH)	*Central Venous Catheters*	*Central Venous Catheter*
	*Embolism, Air*	*Air Embolism*
		*Air Embolisms Gas Embolism*
		*Gas Embolisms*
LILACS (DeCS)	Cateteres Venosos Centrais	*Central Venous Catheter*
	*Central Venous Catheters*	Embolia Gasosa
	*Catéteres Venosos Centrales*	Embolia de Ar
	Embolia Aérea *Embolism, Air*	*Air Embolism*
	*Embolia Aérea*	*Air Embolisms Gas Embolism Gas Embolisms*
		*Embolia Gaseosa*
		*Embolia de Aire*
EMBASE (Emtree)	*central venous catheter*	*AXERA*
	*air embolism*	*Broviac*
		*central intravenous*
		*catheter*
		*central line*
		*central vein catheter*
		*central venous access catheter*
		*central venous access device*
		*central venous catheters*
		*central venous line*
		*cv cath*
		*CVP line*
		*Groshong*
		*Leonard*
		*Leonard catheter*
		*LOGICATH*
		*Orion II*
		*PediaSat*
		*Powerwand*
		*Pro-Line*
		*Secalon-T*
		*short-term central venous*
		*catheterization kit*
		*Vortex Port aero-embolism*
		*aeroembolism*
		*air embolus*
		*embolism air*
CINAHL (*CINAHL Subject Headings*)	*Embolism, Air*	*Air Embolism Gas Embolism Broviac Catheter Broviac Catheters*
	*Central Venous Catheters*	*Catheter Central Venous Central Venous Catheter Hickman Catheter Hickman Catheters*
Web of Science (MeSH)	*Central Venous Catheters Embolism, Air*	*Central Venous Catheter*
		*Air Embolism*
		*Air Embolisms Gas Embolism Gas Embolisms*

Fonte: Elaborada pelos autores.

**Tabela 3:** Estratégia de busca da revisão integrativa, Alfenas, 2024.

**Base de dados**	**Estratégia de busca**
PubMed	((((((“*Central Venous Catheters*”) OR (“*Central Venous Catheter*”)) AND (“*Embolism, Air*”)) OR (“*Air Embolism*”)) OR (“*Air Embolisms*”)) OR (“*Gas Embolism*”)) OR (“*Gas Embolisms*”)
LILACS	(“Cateteres Venosos Centrais”) OR (“*Central Venous Catheters*”) OR (“*Central Venous Catheter*”) OR (“*Catéteres Venosos Centrales*”) AND (“Embolia Aérea”) OR (“Embolia Gasosa”) OR (“Embolia de Ar”) OR (“*Embolism, Air*”) OR (“*Air Embolism*”) OR (“*Air Embolisms*”) OR (“*Gas Embolism*”) OR (“*Gas Embolisms*”) OR (“*Embolia Aérea*”) OR (“*Embolia Gaseosa*”) OR (“*Embolia de Aire*”)
EMBASE	('*central venous catheter*' OR '*axera*' OR '*broviac*' OR '*central intravenous catheter*' OR '*central line*' OR '*central vein catheter*' OR '*central venous access catheter*' OR '*central venous access device*' OR '*central venous catheters*' OR '*central venous line*' OR '*cv cath*' OR '*cvp line*' OR '*groshong*' OR *'leonard*' OR '*leonard catheter*' OR '*logicath*' OR '*orion ii*' OR '*pediasat*' OR 'powerwand' OR '*pro-line*' OR '*secalon-t*' OR '*short- term central venous catheterization kit*' OR '*vortex port*') AND ('*air embolism*' OR '*aero-embolism*' OR '*aeroembolism*' OR '*air embolus*' OR '*embolism air*') AND [embase]/lim
CINAHL	(“*Embolism, Air*” OR “*Air Embolism*” OR “*Gas Embolism*”) AND (“*Central Venous Catheters*” OR “*Broviac Catheter*” OR “*Broviac Catheters*” OR “*Catheter Central Venous*” OR “*Central Venous Catheter*” OR “*Hickman Catheter*” OR “*Hickman Catheters*”)
Web of Science	“*central venous catheters*” (Topic) OR “*central venous catheter*” (Topic) AND “*embolism, air*” (Topic) OR “*air embolism*” (Topic) OR “*air embolisms*” (Topic) OR “*gas embolism*” (Topic) OR “*gas embolisms*” (Topic)

Fonte: Elaborada pelos autores.

Os resultados foram organizados nos softwares EndNote^17^ e Rayyan^18^. Após a exclusão das duplicatas, a seleção dos estudos foi realizada por dois revisores, de forma independente, em duas etapas: inicialmente, procedeu-se à leitura de títulos e resumos, com inclusão dos artigos que respondiam à pergunta de pesquisa; em seguida, realizou-se a leitura na íntegra dos artigos selecionados. Nos casos em que não houve consenso quanto à elegibilidade, um terceiro revisor foi consultado. Ao final, as referências dos estudos incluídos foram avaliadas para identificar possíveis artigos adicionais.

Para a extração de dados, utilizou-se um roteiro elaborado pelos autores, estruturado em um quadro-síntese (Tabela 4) para cada estudo incluído na revisão integrativa. Nesse quadro, foram registrados: título, autores, periódico, país, data, desenho do estudo, nível de evidência, objetivo, método, resultados e conclusão.

**Tabela 4:** Quadro-síntese dos estudos, Alfenas, 2024.

**Título**	**Autores, periódico, país, data**	**Desenho do estudo, nível de evidência**	**Objetivo**	**Método**	**Resultados**	**Conclusão**
Tunnelled haemodialysis catheter removal: an underappreciated problem, not always simple and safe^22^	Tomasz Porazko, Jacek Hobot, Zbigniew Ziembik e Marian Klinger, *International Journal of Environmental Research and Public Health*, Polônia, 27/04/2020.	Estudo retrospectivo, nível de evidência 4	Analisar o uso de um método modificado (*modified cut-down method,* MCDM) para a retirada de cateteres venosos centrais.	Análise retrospectiva de prontuários de 143 pacientes dos Departamentos de Nefrologia e Diálise e de Cirurgia Geral e Vascular do Hospital da Universidade de Opole, com comparação dos resultados dos pacientes submetidos ao *cut-down method* (CDM) ou ao MCDM,	Foi registrado um caso de embolia aérea relacionado à técnica CDM, tratado com sucesso. Não houve casos de embolia aérea entre os pacientes que foram submetidos à técnica MCDM.	A técnica MCDM demonstrou-se mais segura para prevenção da embolia gasosa na retirada de cateteres venosos centrais.
Over-catheter tract suture to prevent bleeding and air embolism after tunnelled catheter removal^23^	Krzysztof Letachowicz, Tomasz Gołębiowski, Mariusz Kusztal, Jan Penar, Waldemar Letachowicz, Wacław Weyde, Marian Klinger, *Journal of Vascular Access*, Polônia, 21/03/2016	Estudo retrospectivo, nível de evidência 4	Demonstrar que a técnica *catheter removal technique with over-catheter suture* apresenta eficácia na prevenção de embolia aérea e sangramento durante a retirada de cateteres venosos centrais.	Avaliação da efetividade de uma técnica de remoção de cateter venoso central, por meio da análise de prontuários de 79 pacientes submetidos ao procedimento em um hospital de Wroclaw, Polônia.	A técnica *catheter removal technique with over-catheter suture* demonstrou maior segurança na prevenção de embolia aérea em comparação à técnica convencional.	A utilização da técnica *catheter removal technique with over-catheter suture* diminui o risco de embolia aérea durante a retirada de cateteres venosos centrais.

Fonte: Elaborada pelos autores.

A extração dos dados foi realizada por dois revisores, de forma independente. Os estudos foram avaliados quanto ao nível de evidência, segundo Melnyk & Fineout-Overholt^19^, e quanto à qualidade metodológica, conforme os critérios propostos pelo McMaster University Occupational Therapy Evidence-Based Practice Research Group, de acordo com o desenho dos estudos incluídos^20^.

## RESULTADOS

A estratégia de busca resultou na recuperação de 2.185 estudos, sendo 752 provenientes do PubMed, 364 do CINAHL, 617 do EMBASE, 274 do LILACS e 178 do Web of Science. Após aplicação dos critérios de inclusão, foram selecionados dois artigos que compuseram a amostra final, conforme ilustrado no fluxograma PRISMA^21^ (Figura 1). A síntese dos dois estudos incluídos está apresentada na Tabela 4.

**Figura 1:** Fluxograma PRISMA. Fonte: adaptado de Page et al.^15^. PRISMA = *Preferred Reporting Items for Systematic Reviews and Meta-Analyses*.

Em 2020, foi realizado na Polônia um estudo retrospectivo com o objetivo de avaliar um método modificado – *modified cut-down method* (MCDM) – para a remoção de CVCs^22^. A população do estudo foi composta por pacientes do Departamento de Nefrologia e Diálise e do Departamento de Cirurgia Geral e Vascular do Hospital da Universidade de Opole. O método consistiu em uma análise retrospectiva de 76 prontuários de pacientes submetidos ao método padrão – *cut-down method* (CDM) – e de 67 prontuários de pacientes submetidos ao MCDM, seguida de comparação entre as técnicas. Como resultado, verificou-se a ocorrência de um caso de embolia gasosa no grupo de pacientes que foram submetidos à técnica CDM, enquanto no grupo submetido ao MCDM não foi registrada tal complicação.

Um estudo retrospectivo realizado na Polônia, publicado em 2016^23^, avaliou uma nova técnica – *catheter removal technique with over-catheter suture*. A população do estudo foi composta por 79 pacientes de um hospital de Wroclaw, Polônia. O método consistiu na análise retrospectiva de 40 prontuários de pacientes submetidos à técnica convencional e de 39 prontuários de pacientes submetidos à nova técnica, seguida de comparação entre ambas. Como resultado, observou-se que a nova técnica apresentou maior eficácia na prevenção de embolia aérea após a retirada do cateter, além de ser mais simples de aplicar e não gerar custos adicionais.

A comparação entre as técnicas descritas nos dois estudos incluídos nesta revisão está apresentada na Tabela 5. Ademais, a avaliação da qualidade dos estudos, realizada conforme os critérios da ferramenta proposta pelo McMaster University Occupational Therapy Evidence-Based Practice Research Group, encontra-se na Tabela 6.

**Tabela 5:** Comparação entre as técnicas propostas nos estudos, Alfenas, 2024.

**Etapas**	***Modified cut-down method***	***Catheter removal technique with over-catheter suture***
Posicionamento do paciente	Trendelenburg	Trendelenburg leve
Assepsia	Utilização da técnica estéril, sem detalhamento	Desinfecção da área e colocação de campos estéreis
Anestesia	Injeção de anestésico local (lidocaína 1%), ao longo da área de incisão	Injeção de anestésico local ao longo da área de sutura e próxima ao *cuff* do cateter
Sutura (antes da remoção do cateter)	Não utilizada	Realiza-se sutura ao redor do cateter, o mais próximo da entrada do cateter na veia jugular interna.
Incisão	Incisão de 2 cm paralela ao cateter	Incisão pequena próxima ao *cuff*
Remoção do cateter	Realiza-se a dissecção dos tecidos com isolamento do cateter. Em seguida, sem seccionar o cateter, a parte intravenosa é retirada da veia cava superior por meio da incisão cutânea. Aplica-se pressão sobre o ponto de entrada da veia por alguns minutos. Após a retirada, o cateter é seccionado distalmente ao *cuff* e essa parte distal é retirada pelo sítio de saída.	Realiza-se o isolamento do cateter e sua exposição com o auxílio de um fórceps cirúrgico. O *cuff* é dissecado e a porção intravascular é seccionada. Em seguida, essa porção é removida enquanto o paciente mantém a respiração em apneia.
Hemostasia	Realiza-se hemostasia.	Não mencionado
Síntese	Fechamento da incisão com duas a três suturas não absorvíveis com fios 3.0	Sutura não absorvível ao longo da antiga região de passagem do cateter, enquanto se aplica uma leve pressão na veia jugular interna. São feitas suturas adicionais na região do *cuff* removido.
Curativo	Após a síntese, é aplicado um curativo sobre a ferida cirúrgica. Não é especificado o tempo de retirada das suturas.	Após a síntese, realiza-se nova desinfecção do sítio cirúrgico e aplica-se um curativo sobre a ferida. As suturas são retiradas após 72 h.

Fonte: Elaborada pelos autores.

**Tabela 6:** Avaliação da qualidade metodológica dos estudos quantitativos, Alfenas, 2024.

**Revisão crítica dos estudos quantitativos**		**Estudo 1** ^22^	**Estudo 2** ^23^
Objetivo do estudo	O objetivo estava claro? (sim/não)	Sim	Sim
Literatura	Foi realizada uma revisão da literatura relevante sobre esse tema?	Não	Sim
Desenho	Randomizado/coorte/estudo de caso único/antes e depois/ caso-controle/transversal/ estudo de caso/longitudinal	Sim	Sim
Amostra	A amostra foi descrita detalhadamente? (sim/não)	Sim	Não
Amostra	Foi apresentada justificativa para o tamanho da amostra? (sim/não/não se aplica)	Não	Não
Desfechos	As medidas dos desfechos eram confiáveis? (sim/não/não informado)	Sim	Não informado
Desfechos	As medidas dos desfechos eram válidas? (sim/não/não informado)	Sim	Sim
Intervenção	A intervenção foi descrita de forma detalhada? (sim/não/não informado)	Sim	Sim
Intervenção	A contaminação foi evitada? (sim/não/não informado/não se aplica)	Sim	Sim
Intervenção	A intervenção simultânea foi evitada? (sim/não/não informado/não se aplica)	Não informado	Não informado
Resultados	Os resultados foram relatados em termos de significância estatística? (sim/não/não se aplica/não informado)	Sim	Não
Resultados	Os métodos de análise foram adequados? (sim/não/não informado)	Sim	Não informado
Resultados	A importância clínica foi relatada? (sim/não/não informado)	Sim	Sim
Resultados	Houve relato de participantes que abandonaram o estudo? (sim/não)	Não	Não
Conclusões	As conclusões foram coerentes com os métodos e resultados do estudo? (sim/não)	Sim	Sim

Fonte: elaborada pelos autores.

## DISCUSSÃO

Observou-se nesta revisão a escassez de estudos que documentem a efetividade das técnicas de remoção de CVCs, sendo possível analisar apenas dois artigos, ambos realizados na Polônia, com desenho retrospectivo e nível de evidência classificado como quatro, e voltados à remoção de cateteres de longa permanência^22,23^. Esse cenário reforça a necessidade de novos estudos com delineamento experimental conduzidos em ambientes clínicos.

Os dois estudos incluídos nesta revisão documentam técnicas distintas para a remoção de CVCs, visando à prevenção de embolia gasosa. A técnica MCDM, descrita em 2020, surgiu como uma modificação do método convencional (CDM)^22^. No MCDM, realiza-se uma incisão e divulsão dos tecidos para localização e liberação do *cuff* do cateter tunelizado. Em seguida, procede-se à retirada da parte intravascular, com hemostasia por compressão local, e então é feito o corte acima do *cuff*. Por fim, a extremidade do cateter é removida pelo orifício original de inserção do cateter, sendo realizados a sutura e os curativos. O diferencial em relação ao CDM é que, no MCDM, o corte acima do *cuff* é feito antes da retirada da parte intravascular. De acordo com o estudo, no grupo submetido ao CDM ocorreu um caso de embolia gasosa (1,3%), enquanto no grupo submetido ao MCDM não houve essa complicação, sugerindo que a técnica modificada pode ser eficaz na prevenção da embolia aérea associada à retirada de CVCs^22^.

A técnica de remoção com sutura sobre cateter (*catheter removal technique with over-catheter suture*), relatada em 2016^23^, baseia-se nos seguintes passos: o paciente é colocado em posição de Trendelenburg leve, realiza-se a antissepsia da área de inserção do cateter e procede-se ao seu isolamento com campos estéreis. Na sequência, é feita a infiltração local com anestésico e posiciona-se uma sutura ao redor da veia jugular interna, no ponto de inserção do cateter, proximal ao *cuff*. Posteriormente, realiza-se uma incisão na pele próxima ao *cuff*, que é isolado e exposto com auxílio de uma pinça cirúrgica. O *cuff* é exposto e o cateter, clampeado acima dele. Em seguida, o cateter é cortado e seu segmento intravascular é removido enquanto o paciente realiza apneia com retenção do ar. A sutura previamente posicionada é então amarrada sobre o antigo trajeto da veia jugular interna, no local onde se encontrava a parte intravascular do cateter, aplicando-se leve compressão manual. Suturas adicionais são realizadas na área da incisão, seguidas de nova antissepsia do local e realização dos curativos. As suturas costumam ser removidas 72 horas após o procedimento.

Registra-se que os métodos de investigação empregados nos estudos não permitem afirmar a superioridade de uma técnica sobre a outra na prevenção de embolia gasosa durante a remoção de CVCs. Ademais, não foram identificados na literatura estudos com maior rigor metodológico que comparem a efetividade das técnicas, embora existam descrições de alguns relatos de caso^24-26^.

Como exemplo, relata-se o caso de um paciente do sexo masculino, de 66 anos, submetido a colocação de um cateter de diálise de curta permanência e alto fluxo na veia jugular interna direita para realização de plasmaférese devido a injúria renal aguda^24^. Em decorrência de mau funcionamento da cama hospitalar, o paciente teve o cateter removido com a cabeceira levemente elevada, em posição supina. Pouco tempo depois, apresentou dispneia grave, hemiparesia, taquicardia, hipóxia e hipotensão, sendo diagnosticado com embolismo gasoso cerebral. O tratamento incluiu oxigênio 100% e posicionamento em Trendelenburg, com boa recuperação^24^. Esses achados sugerem que o posicionamento do paciente é um fator relevante na prevenção da embolia gasosa, uma vez que a entrada de ar ocorre por pressão negativa no cateter. Ademais, conforme a opinião de especialistas^27^, tal cuidado, juntamente com a oclusão imediata do orifício no momento da retirada do cateter, tem sido recomendado na literatura para evitar a embolia gasosa. Ressalta-se, entretanto, que, embora esses relatos discutam tais medidas profiláticas, seu nível de evidência não permite a generalização dos resultados.

Outro relato de caso, descrito em 2023, também evidencia a importância do posicionamento do paciente durante a retirada do CVC^25^. Nesse relato, um paciente de sexo masculino, de 77 anos, internado por diverticulite perfurada do sigmoide, teve um cateter de baixo fluxo e curta permanência removido da veia jugular interna direita enquanto estava em posição sentada. Imediatamente após o procedimento, apresentou hipotensão arterial, diminuição do nível de consciência, dor localizada e hemiparesia esquerda. Exames de imagem documentaram bolhas nos seios cavernosos e nas cisternas da base; o paciente foi submetido a tratamento, e as bolhas foram reabsorvidas. O diagnóstico de embolismo aéreo retrógrado na via central reforça a importância de realizar o procedimento com o paciente em posição horizontal no leito^25^. Ressalta-se, entretanto, que não foram encontrados estudos que comparem a posição mais segura para a remoção do CVC.

Em 2013, foi descrito um caso de formação de bainha composta por fibrina ao redor de cateteres, especialmente os de longa permanência^26^. Trata-se de uma mulher de 75 anos, portadora de cardiomiopatia hipertensiva com episódios de insuficiência cardíaca e fibrilação atrial, internada em unidade de terapia intensiva para monitoramento após cirurgia de remoção da glândula parótida direita. Durante a admissão, exames de imagem evidenciaram posicionamento incorreto do CVC e diagnosticaram oclusão trombótica completa da veia jugular interna esquerda, indicando a necessidade de remoção do cateter. A paciente estava em ventilação mecânica e foi posicionada em Trendelenburg. Durante o procedimento, foi encontrada resistência à remoção do cateter; imediatamente após, foi aplicada pressão local com gaze para controle de sangramento. Posteriormente, a paciente desenvolveu instabilidade hemodinâmica. O ecocardiograma evidenciou microbolhas de ar nas câmaras cardíacas direitas, sincronizadas com a respiração e aparentemente originadas da veia cava superior. Tomografia computadorizada de pescoço e tórax demostrou oclusão trombótica total da veia jugular interna esquerda, estendendo-se até o tronco braquiocefálico e veia subclávia esquerda, com presença de bolhas de ar em seu interior. O diagnóstico de embolia venosa aérea foi associado à remoção do CVC. Uma segunda ultrassonografia vascular evidenciou um trajeto entre a veia e a derme, responsável pela embolia gasosa, visível mais de 24 horas após a colocação do curativo oclusivo^26^.

O relato sugere que tratos de fibrina podem se formar ao redor do cateter, especialmente nos de longa permanência, aumentando o risco de embolia gasosa após a remoção de CVC ao criar um trajeto para entrada de ar. Desse modo, técnicas que ocluam esses tratos imediatamente após a remoção do cateter podem reduzir a incidência de embolia gasosa aérea associada ao procedimento.

Portanto, estratégias como a técnica de sutura sobre o cateter^21^ podem ser úteis para prevenir a embolia gasosa causada pela persistência do trajeto entre a derme e a veia. É importante considerar que não foi identificado nenhum estudo que determine a melhor técnica de curativo após a remoção do cateter, nem o tempo necessário para a oclusão completa do orifício.

O artigo publicado em 2016 descreve a retirada do cateter com o paciente realizando a retenção de ar^20^, enquanto o estudo de 2020 não relata essa intervenção^20^. Embora não tenham sido identificadas pesquisas que avaliem a efetividade dessa prática na remoção do cateter, um estudo de 2001 comparou a pressão venosa central em diferentes manobras realizadas durante a inserção do CVC^28^. Foram avaliadas três situações: paciente falando, em apneia e realizando a manobra de Valsalva. Foram mensurados os valores de pressão venosa central, e constatou-se que a manobra de Valsalva foi a que mais elevou a pressão venosa central e gerou menor porcentagem de pressão intravascular negativa nos 40 pacientes avaliados (2,5% = 1/40)^28^. Dessa forma, a adoção da manobra de Valsalva durante a remoção do cateter pode ser uma medida eficaz para a prevenção dessa complicação.

Dada a gravidade da embolia gasosa, algumas medidas de tratamento têm sido documentadas na literatura^29^, incluindo oxigenação do paciente a 100% em posição de Trendelenburg, aspiração via cateter ou uso de câmara hiperbárica. Também é mencionada a antibioticoterapia empírica com cobertura para *Staphylococcus aureus* e *S. epidermidis*. Considerando que o tratamento foge ao escopo desta revisão, sugere-se a consulta a outras revisões para avaliar a efetividade dessas medidas.

Entre as limitações dos estudos apresentados, destacam-se o caráter retrospectivo das análises e o tamanho reduzido das amostras de pacientes. Não foram identificados estudos que analisem a remoção de cateteres de curta permanência e baixo fluxo. Foram excluídos da revisão estudos secundários, relatos de caso, literatura cinzenta, cartas-resposta e editoriais. Apesar dessas limitações, os estudos apresentam alternativas promissoras para redução de danos ao paciente, como a padronização dos procedimentos e a adoção de novas técnicas. Contudo, as intervenções propostas devem considerar as diferentes realidades socioculturais e os custos envolvidos na prestação dos cuidados durante a remoção do cateter.

## CONCLUSÃO

Por meio desta revisão, verificou-se que há poucos estudos que descrevam as melhores evidências científicas para a prevenção da embolia gasosa durante a remoção de CVCs em adultos. Dois estudos relatam técnicas que podem ser benéficas: o método MCDM e a técnica *catheter removal technique with over-catheter suture*. Contudo, novos estudos experimentais são necessários para determinar qual técnica apresenta melhores resultados na prevenção da embolia gasosa.

Em contrapartida, não é possível assegurar quais dos procedimentos comumente adotados na prática clínica para a remoção de cateteres — como posicionamento do paciente em Trendelenburg, realização da manobra de Valsalva e aplicação de curativos compressivos no sítio de remoção — representam as medidas mais eficazes para a prevenção da embolia. Dessa forma, são necessários mais estudos que avaliem a efetividade dessas intervenções.

Assim, esta revisão evidencia a necessidade de maior discussão sobre o tema, considerando que as consequências da embolia gasosa relacionada à remoção do CVC podem ser muito prejudiciais aos pacientes e representam um efeito indesejado da assistência à saúde. Além disso, a falta de padronização da retirada dos cateteres favorece práticas intuitivas, que não garantem segurança no procedimento e podem resultar em iatrogenias.

## Data Availability

Data sharing does not apply to this article, as no data were generated or analyzed.
